# Supplementary material for: Use of oral anticoagulants in older people with atrial fibrillation in UK general practice: protocol for a cohort study using the Clinical Practice Research Datalink (CPRD) database
Source: BMJ Open. 2019 Dec 15;9(12):e032646. doi: 10.1136/bmjopen-2019-032646 (PMC6924720; doi:10.1136/bmjopen-2019-032646)
Supplement: Author's manuscript [file bmjopen-2019-032646.draft_revisions.pdf]

BMJ Open is committed to open peer review. As part of this commitment we make the peer review history of every article we publish publicly available.

When an article is published we post the peer reviewers' comments and the authors' responses online. We also post the versions of the paper that were used during peer review. These are the versions that the peer review comments apply to.

The versions of the paper that follow are the versions that were submitted during the peer review process. They are not the versions of record or the final published versions. They should not be cited or distributed as the published version of this manuscript.

BMJ Open is an open access journal and the full, final, typeset and author-corrected version of record of the manuscript is available on our site with no access controls, subscription charges or pay-per-view fees (http://bmjopen.bmj.com).

If you have any questions on BMJ Open's open peer review process please email info.bmjopen@bmj.com

BMJ Open

Utilisation of oral anticoagulants in older people with atrial fibrillation in UK general practice: a protocol for a cohort study using the Clinical Practice Research Datalink (CPRD) database

Journal:	*BMJ Open*
Manuscript ID	bmjopen-2019-032646
Article Type:	Protocol
Date Submitted by the Author:	28-Jun-2019
Complete List of Authors:	Mitchell, Anneka; University of Bath, Pharmacy and Pharmacology; University Hospital Southampton NHS Foundation Trust, Pharmacy Research Centre<br>Welsh, Tomas; Research Institute for the Care of Older People; University of Bristol, Institute of Clinical Neurosciences<br>Watson, Mags; Strathclyde Institute of Pharmacy and Biomedical Sciences<br>Snowball, Julia; University of Bath, Pharmacy and Pharmacology<br>McGrogan, Anita; University of Bath, Pharmacy and Pharmacology
Keywords:	Atrial fibrillation, Anticoagulants, Stroke < NEUROLOGY, Aged, Observational study

SCHOLARONE™
Manuscripts

UTILISATION OF ORAL ANTICOAGULANTS IN OLDER PEOPLE WITH ATRIAL FIBRILLATION IN UK GENERAL PRACTICE: A PROTOCOL FOR A COHORT STUDY USING THE CLINICAL PRACTICE RESEARCH DATALINK (CPRD) DATABASE

Anneka Mitchell[1, 2], Tomas Welsh[3, 4], Margaret C Watson[5], Julia Snowball[1] and Anita McGrogan[1]

1. Department of Pharmacy and Pharmacology, University of Bath, Bath, BA2 7AY, UK

2. Pharmacy Research Centre, University Hospital Southampton, Southampton, SO16 6YD, UK

3. Research Institute for the Care of Older People (RICE), Bath, BA1 3NG, UK

4. University of Bristol, Bristol, BS8 1TH, UK

5. Strathclyde Institute of Pharmacy and Biomedical Sciences, University of Strathclyde, Glasgow, G4 0RE, UK

Corresponding author: Mrs Anneka Mitchell, Department of Pharmacy and Pharmacology, University of Bath, Bath, UK. BA2 7AY. Email: Anneka.mitchell@bath.ac.uk Tel: 01225 384215

Word count: 4408

For peer review only

For peer review only - http://bmjopen.bmj.com/site/about/guidelines.xhtml

ABSTRACT

Introduction

Warfarin has frequently been under-used in older people for stroke prevention in atrial fibrillation (AF). Direct oral anticoagulants (DOACs) entered the UK market from 2008 and have been recommended as an alternative to warfarin. This study aims to describe any changes in the prescribing of OACs to people aged ≥ 75 years in UK general practice before and after the introduction of DOACs; examine differences in patient characteristics which may influence prescribers' decisions regarding anticoagulation; to evaluate the time people stay on OACs and switching between OACs.

Methods and analysis

Patients with a diagnosis of AF will be identified from the Clinical Practice Research Datalink (CPRD). The study period will run from 1st January 2003 to 27th December 2017. Patients enter the cohort at the latest date of: start of study period, first AF diagnosis, 75th birthday or a year from when they started to contribute research standard data. Follow-up continues until they leave the practice, death or the date the practice stops contributing research standard data. Exposure to OACs will be defined as ≥ 1 prescription issued for an OAC of interest during the study period. Patients entering the study on an OAC and those issued an OAC in the year preceding study entry will be defined as "prevalent users". Patients starting on an OAC during the study period will be defined as "incident users". Incidence and prevalence of OAC prescribing, patient demographics and characteristics will be described during three time periods: 2003-2007, 2008-2012, and 2013-2017. Persistence (defined as time from initiation to discontinuation of medication) with and switching between different OACs will be described.

Ethics and Dissemination

The protocol for this study was approved by the CPRD Independent Scientific Advisory Committee. The results will be disseminated in a peer-review journal and at conferences.

Trial registration number

EUPAS29923

Keywords

Atrial fibrillation, Anticoagulants, Stroke, Aged, Observational study

For peer review only

For peer review only - http://bmjopen.bmj.com/site/about/guidelines.xhtml

ARTICLE SUMMARY

Strengths and limitations of this study

- This will be a large cohort study using a validated data source which is representative of the UK population.
- This study will explore a number of aspects of OAC prescribing and examine changes over time to show any changes in OAC prescribing practices since the introduction of DOACs.
- Analysing differences in patient demographics, co-morbidities and co-prescribing between those prescribed DOACs and warfarin will provide important information for future studies aiming to compare differences in outcomes with the different OACs.
- This study relies on prescription data generated by general practices, therefore we will not know if the prescribed medication was ever dispensed or taken.
- Warfarin dosing schedules are not recorded in the CPRD so duration of warfarin prescriptions will be estimated.

INTRODUCTION

A large body of evidence has shown that vitamin K antagonists are safe and effective for stroke prevention in atrial fibrillation (AF), but they have frequently been under-used in older people.[1] Reasons cited by physicians as making them less likely to prescribe vitamin K antagonists for a person with AF include advancing age, risk of falls, co-morbidities and previous bleeding.[2]

The first direct oral anticoagulant (DOAC), dabigatran, was marketed throughout Europe and in the UK in 2008, but was not licensed for stroke prevention in AF until 2011. Since then, three additional DOACs have been licensed for prevention of stroke: rivaroxaban, apixaban and edoxaban, and these are recommended in national and international guidelines as an alternative to warfarin.[3-5] In the UK, the National Institute of Clinical Excellence (NICE) produces technology appraisals making recommendations for new medicines. The National Health Service (NHS) is legally obliged to fund those medicines recommended by NICE within 3 months of publication of the appraisal. NICE published favourable technology appraisals for dabigatran and rivaroxaban in 2012,[6 7] apixaban in 2013,[8] and edoxaban in 2015[9] meaning DOACs were available across the UK from mid-2012 onwards for stroke prevention in AF.

DOACs may allay some prescribers' concerns when deciding whether to anticoagulate their older patients: they have fixed dosing schedules and can be added to compliance aids. They have also been shown in clinical trials to have a significantly lower risk of intracranial haemorrhage than warfarin[10-13] (a prominent concern in patients with a history of falls).[14]

Since the introduction of DOACs, the overall rate of OAC initiation has increased by 58%.[15] However, it is not known whether the introduction of DOACs has changed the rates of OAC prescribing for older people (aged ≥ 75 years) or how patient demographics, co-morbidities and concomitant prescribing affect the choice of anticoagulant prescribed in UK general practice.

For peer review only - http://bmjopen.bmj.com/site/about/guidelines.xhtml

This study aims to characterise prescribing of OACs to people aged ≥ 75 years in UK general practice, before and after the introduction of DOACs.

Objectives

1. To describe changes in the point prevalence and incidence of OAC prescribing by year prior to the introduction of DOACs (2003 – 2007), between the introduction of DOACs and the time they were recommended by NICE for stroke prevention in AF (2008 – 2012), and following NICE recommendation (2013 – 2017).

2. To describe switching between OACs during the study period.

3. To compare the characteristics of patients with AF who were newly started on OACs during each period described in objective 1, to those who were not prescribed anticoagulation in the same period.

4. To describe persistence with DOACs compared with warfarin.

METHODS AND ANALYSIS

This will be a retrospective cohort study using routinely collected healthcare data from the Clinical Practice Research Datalink (CPRD) database. The CPRD contains anonymised medical records and prescribing data from general practitioners in primary care. It contains data for around 7% of the UK population and is representative in age, sex and ethnicity.[16] Sociodemographic data, medical diagnoses, other clinical and test data are recorded using Read codes and these Read codes will be used to identify patients to be included in the study and also to identify covariates as described below.

Source population

The study period will start on the 1st January 2003 and end 27th December 2017. The source population will consist of all patients in CPRD who have ≥ 1 Read codes for AF and have contributed a minimum of 12 months research standard data prior to entry in to the cohort. To be eligible to join the study, patients need to have the following: a Read code for AF recorded during or any time prior to the start of the study period; evidence to support the diagnosis of AF such as a second AF diagnosis at a later date, an echocardiogram or prescription for a rate limiting or antiarrhythmic medication in the 3 months before the AF diagnosis or any time after; and be aged ≥ 75 years at study entry.

Patients will enter the cohort at the latest of the following dates:

- Start of study period
- First AF diagnosis
- 75th birthday
- A year from the date they started to contribute research standard data

Study periods

The cohort will be analysed in three time periods:

- Period 1: prior to the introduction of DOACs (2003-2007)

For peer review only - http://bmjopen.bmj.com/site/about/guidelines.xhtml

- Period 2: during the period between the introduction of DOACs and the time they were recommended by NICE (2008 – 2012)
- Period 3: following the publication of the NICE technology appraisals recommending DOACs as an option for stroke prevention in AF (2013 – 2017).

For each time period, patients will contribute data to either the warfarin, DOAC or no OAC group depending on whether they were prescribed an OAC during that period. Patients in the cohort can contribute data to more than one time period and more than one group, for example they may enter the study during the first time period but not be prescribed an OAC until period 3. In this case they would contribute data to the no-OAC group in time period 1 and 2 and then one of the OAC groups in period 3.

Patients will be censored if they leave the practice, the practice stops contributing data, at their date of death, or at the end of the study. When analysing incidence of OAC prescribing and when comparing characteristics of patients prescribed OACs, patients will also be censored if they are prescribed an OAC.

Exposure

The OACs of interest in this study are warfarin, dabigatran, rivaroxaban, apixaban, and edoxaban. Warfarin is the only vitamin K antagonist (VKA) included as it is the most commonly used VKA in the UK. Dabigatran, rivaroxaban, apixaban and edoxaban are the only DOACs licensed for the prevention of stroke in AF available during the study period.

All prescriptions for the OACs of interest that are issued during the study period and in the year prior to entry in to the study cohort will be identified.

Incident and prevalent users

The index date for exposure to an OAC will be the date of the first OAC prescription within the study period. Patients prescribed an OAC in the year prior to entry into the cohort will be classed as “prevalent users”. Patients started on an OAC during the study period with ≥ 1 year free of OAC prescriptions will be defined as “incident users”.

Indication for prescribing

OACs are prescribed for many different indications but only those prescribed for stroke prevention in AF will be included in this study. Patient records will be investigated further if they meet ≥ 1 of the following criteria to ascertain if the OAC was commenced for AF or for another indication:

- They had a hip fracture or hip replacement in the 6 weeks prior to the OAC index date
- They had a knee replacement in the 2 weeks prior to the OAC index date
- They had a venous thromboembolism in the 6 months prior to the OAC index date

DOAC prescription duration

The start date will be defined as the date the first prescription was issued. The expected end date will be calculated by dividing the number of tablets prescribed by the licensed number of doses per day (i.e. 2 doses per day for dabigatran and apixaban and 1 dose per day for rivaroxaban and edoxaban). Where the quantity of tablets prescribed is not specified, the

For peer review only - http://bmjopen.bmj.com/site/about/guidelines.xhtml

expected duration will be imputed from other prescriptions issued for the same product for that patient, or overall for that product if patient specific information is not available.

Patients will be classified as being continuously exposed until there is a gap of > 30 days between the expected end of one prescription and the start of the next. Where there is a gap of ≤ 30 days between the expected end of one prescription and the start of another for the same DOAC (regardless of whether the strength prescribed changes) the gap will be filled and defined as continuous exposure. Where a subsequent prescription for the same product is issued before the expected end of the previous prescription the prescriptions will be concatenated and the extra days added to the expected end date of the second prescription. A maximum of 30 additional days will allowed per patient.

A DOAC will be defined as discontinued 60 days from the expected end date of the last prescription issued for that product.

Warfarin prescription duration

Warfarin does not have a fixed dosing schedule; the dose is amended based on the results of a blood test, the international normalised ratio (INR). In the UK, patients are issued with different strength tablets (0.5mg, 1mg, 3mg and 5mg) and are advised, following each INR test, what dose they need to take and what strength of tablets to use. Dosing information for warfarin is not recorded in the CPRD, however, the strength and quantity of warfarin tablets issued by the general practice are recorded. Read codes for warfarin monitoring and results of INR tests are also recorded by some general practices but this is not recorded consistently.

The index date will be defined as either the date of the first prescription issued, or from the date of the first INR test result above 2 if this occurs before the first issue of a prescription. Patients may be exposed to warfarin before the first prescription issued by their general practitioner (GP) if treatment is started in hospital. CPRD data does not include hospital prescribing, hence INR test results ≥ 2 will be used as a surrogate marker of exposure.

The expected end date for warfarin prescriptions will be estimated using the median time between all previous prescriptions issued to the same patient. For example, if four prescriptions had been issued for 28 x 1mg tablets and the gaps between the prescription issue dates were 15, 35, and 42 days we would define 28 tablets as lasting 35 days so the expected end date of the last prescription would be 35 days from the issue date. Where multiple strengths are issued for a patient, the median will be calculated for each strength and the expected end date will be the latest of these dates.

If only one prescription has been issued the expected end date will be assumed to be equal to the median warfarin prescription gap for the cohort.

A patient will be assumed to have discontinued warfarin treatment if there is a period ≥ 2 times their median prescription gap. If an INR test result ≥ 2 is recorded in the discontinuation period (expected end date of last prescription to the date equal to twice their median prescription gap), then INR tests will be used as a surrogate marker for warfarin prescribing and the end date will be defined as one week following the final INR test result ≥ 2.

Restarting index OAC after discontinuation

For peer review only - http://bmjopen.bmj.com/site/about/guidelines.xhtml

There could be instances where OAC therapy is classed as discontinued when in fact the OAC has been continued but not issued by a GP if the patient is hospitalised. In instances where patients are classified as discontinuing OAC therapy but have then been “restarted” on their index OAC, they will be reviewed individually to ascertain whether the OAC therapy had truly been discontinued or whether their records indicate another reason for the gap in prescribing e.g. a record indicating an admission to hospital.

OAC Switching

The first OAC prescribed during the study (for incident users) or upon entry to the study (for prevalent users) will be defined as the index OAC. If ≥ 1 prescription for an alternative OAC is prescribed during the study period, the patient will be designated as having switched. Switches can be from:

- Warfarin to DOAC
- DOAC to warfarin
- DOAC to alternative DOAC

Patients will be assumed to have stopped the index OAC on the date the alternative OAC is issued if this is prior to the expected end date of the index OAC. Where the alternative OAC is prescribed after the expected end date of the index OAC, the time to switch will be described.

Covariate classification

It is recognised that some patients may remain in the cohort for a long time and their covariates may change. Therefore covariates will be identified separately for each of the time periods the patient contributes data and be grouped by DOAC, warfarin or no OAC depending on exposure for that period.

Patients will be classified as having a co-morbidity if the patient record contains ≥ 1 diagnostic Read code for a disease or ≥ 3 Read codes supporting the presence of the disease at any time prior to the relevant date for that period. Concomitant medication will be classified as ≥ 1 prescription issues for a medication of interest in the 3 months prior to or following the relevant date for that period.

The relevant date will be the study entry date for the first period the patient contributes data to, the start of the subsequent study period if they remain in the study and do not receive an OAC or the OAC index date if one is prescribed.

For example, a patient may not have dementia when they enter the study in period 1 but may have developed dementia by the time they are prescribed an OAC in period 3. The covariate data used for period 1 would be based on all Read codes dated before they entered the study, the data contributed to period 2 would relate to their Read codes before the start of period 2 and the data contributed to period 3 would relate to their Read codes before their OAC index date.

Definitions of covariates

Differences in patient demographics (age, biological sex) and characteristics (body mass index (BMI), smoking status and alcohol consumption) between those anticoagulated for AF

For peer review only - http://bmjopen.bmj.com/site/about/guidelines.xhtml
For peer review only

and those not anticoagulated will be described for each period. Differences in the stroke risk score (CHA_2DS_2-VASc)[17] and the bleeding risk score (HAS-BLED),[18] co-morbidities and concomitant medication will also be described. These covariates are defined in the following subsections.

CHA_2DS_2-VASc score

International guidelines recommend using this score to calculate stroke risk in patients with AF.[3-5] Higher scores indicate an increasing risk of stroke and the maximum score is 9. Definitions for each element were based on those described by Lip and colleagues[17] but have been modified for clarity:

- *Congestive heart failure (1 point)*
  Right or left ventricular failure or heart failure. Supportive information includes Read codes for heart failure clinic attendance or referral, heart failure monitoring or reference to heart failure therapy/education.
- *Hypertension (1 point)*
  Primary or secondary hypertension or hypertensive disease states e.g. hypertensive heart disease. Supportive information includes codes for monitoring, referral to specialist clinics or reference to therapy for hypertension.
- *Age ≥ 75 (2 points)*
- *Diabetes mellitus (1 point)*
  Type 1 or Type 2 diabetes mellitus diagnoses or the consequences of diabetes mellitus e.g. diabetic retinopathy. This also includes anyone prescribed oral hypoglycaemic agents and/or insulin. Excludes gestational diabetes and steroid induced diabetes as these are often reversible. Supportive information includes dietary advice, reference to diabetes therapies, referral to specialist clinics or educational programs.
- *Stroke/transient ischaemic attack or thromboembolism (2 points)*
  Stroke: Includes ischaemic stroke transient ischaemic attacks. Excludes haemorrhagic stroke, and venous infarctions.
  Thromboembolism: Includes arterial embolic disease and surgeries to treat arterial emboli, excludes thrombosis and venous embolic disease which are included in vascular disease.
- *Vascular disease (1 point)*
  Includes: Coronary artery disease (prior myocardial infarction, angina pectoris, percutaneous coronary intervention or coronary bypass surgery and aortic plaque); Peripheral vascular disease (intermittent claudication, previous surgery or percutaneous intervention on the abdominal aorta, bifurcation or the lower extremity vessels (limited to surgery on the femoral, popliteal, peroneal and iliac arteries)); arterial and venous thrombosis. Excludes thromboses related to pregnancy.
- *Age 64-74 (1 point)*
- *Female sex (score 1)*

HAS-BLED [18]

International guidelines recommend using this score to assess the risk of bleeding with anticoagulants.[3-5] Higher scores indicate an increased risk of bleeding with anticoagulants. The presence of each disease scores 1 point and the maximum score is 7. For the purpose of

For peer review only - http://bmjopen.bmj.com/site/about/guidelines.xhtml

this study, the maximum score for the HAS-BLED will be 6. The L in HAS-BLED corresponds to labile INR defined as a time in therapeutic range with warfarin of less than 60%. As INRs are not recorded consistently in the CPRD and because people prescribed DOACs would not have INR tests at all, this element of the score will not be included. Definitions for each element were based on those described by Pisters and colleagues,[18] but have been adapted to suit the information recorded in the CPRD.

- *Hypertension (1 point)*
  As described for CHA_2DS_2-VASc score above
- *Abnormal renal or hepatic function (1 point)*
  Renal and hepatic function will be determined using a combination of clinical Read codes and test results.
  - Renal impairment:
    Includes chronic dialysis, transplant, chronic renal impairment (where defined as stage 3 or above, moderate, severe or end-stage). If renal impairment is recorded with no severity, but there is a test result for creatinine ≥ 200µmol/L the patient will be allocated a point. If there are no test results recorded the patient will score zero. Codes for renal disease where it does not state that renal function is impaired will be excluded.
  - Hepatic disease:
    Includes chronic hepatic disease (cirrhosis, hepatitis, fatty liver, and fibrosis), liver transplantation, or biochemical evidence of significant hepatic derangement (bilirubin > 2 x upper limit of normal in association with aspartate aminotransferase/alanine aminotransferase/alkaline phosphatase) where these blood test results are recorded in CPRD.
- *Stroke (1 point)*
  As described for CHA_2DS_2-VASc score above
- *Bleeding history or predisposition (1 point)*
  Includes any previous bleed. Categorised based on the International Society of Thrombosis and Haemostasis guidance as major bleeding where the bleed occurs in a critical area or organ or requires a blood transfusion (CPRD does not state the number of units infused so any requirement for a blood transfusion in conjunction with a Read code for bleeding will be classified as a major bleed);[19] or clinically relevant non major bleeding (defined as a bleed not fitting the major bleed criteria but requiring review by a healthcare professional, as the codes are recorded by a GP we will assume that these bleeds required review).[20] Excludes codes for traumatic bleeding, bleeding related to pregnancy and post-operative bleeding. Predisposition to bleeding includes anaemia and gastric, duodenal and peptic ulcers.
- *Elderly (1 point)*
  Defined as being aged > 65 years old
- *Drugs or Alcohol (1 point)*
  A point will be allocated where a patient receives a prescription for an antiplatelet or non-steroidal anti-inflammatory (NSAID) medication within the 3 months before the relevant date for that period. Exact alcohol consumption is not well documented in the CPRD but patients will score a point for this element if they have any record stating the number of units consumed per week as > 8 units or if they have any codes for heavy/problem drinking.

For peer review only - http://bmjopen.bmj.com/site/about/guidelines.xhtml

Renal Impairment

DOACs are predominantly renally cleared and so require dose adjustment and careful monitoring in renal impairment. The elimination of warfarin is mostly metabolic, being metabolised by hepatic enzymes so is not affected by changes in renal function. Renal impairment may therefore affect the decision of which anticoagulant (if any) to use so has been included as a covariate alone, in addition to its inclusion in the HAS-BLED score. The Read code or test result dated closest to the relevant date for the period will be used to categorise the presence and level of renal impairment.

- *Chronic renal impairment*
  Includes chronic dialysis, renal impairment, and chronic kidney disease regardless of whether severity or stage is reported. Test results for creatinine and estimated glomerular filtration rate (eGFR) will also be obtained where available. If test results are available, they will be used to classify whether a patient has renal impairment and the stage of impairment (if eGFR is documented). Codes for a renal disease that may reduce kidney function but with no test or diagnostic code to support the presence of renal impairment will be excluded.
- *Acute kidney injury*
  Previous episodes of acute kidney injury may influence the decision to prescribe an anticoagulant, and which anticoagulant to prescribe. Read codes for acute kidney injury will be counted as discrete events where they occur ≥ 1 week apart.

Hepatic disease

Hepatic disease is an independent risk factor for bleeding and also affects the metabolism of warfarin so may affect the decision to prescribe anticoagulation. Hepatic disease will be defined as specified under HAS-BLED above.

Previous bleed

Includes any non-traumatic bleeding or haemorrhage. Bleeds will be divided in to subsections of major bleeding and clinically relevant non major bleeding (as described in HAS-BLED section), and also by site of bleed (intracranial bleeds, gastrointestinal bleeds, other).

Dementia

Include dementia (any type), Alzheimer's disease, and anyone prescribed an acetylcholinesterase inhibitor or memantine. Excludes cognitive impairment where there is no diagnosis of dementia.

History of or at risk of falls

Includes any type of fall and any Read code which specifically indicates the practitioner is concerned that the patient is at risk of falling. Excludes gait disturbances and other factors which may increase risk of falling but have not been defined by the GP as causing concern regarding falls risk.

Fragility fractures

Fragility fractures are defined by the National Institute of Clinical Excellence (NICE) as "…most commonly in the spine (vertebrae), hip (proximal femur) and wrist (distal radius).

For peer review only - http://bmjopen.bmj.com/site/about/guidelines.xhtml

They may also occur in the arm (humerus), pelvis, ribs and other bones".[21] It is often not recorded in CPRD whether a fracture is thought to be a fragility fracture or not. Read codes for a diagnosis of fracture or operation to repair a fracture occurring in the spine, proximal femur, long bones, distal radius, humerus, pelvis or ribs will be included. Non-specific codes for osteoporotic and fragility fractures will also be included. Pathological fractures (unless specified that they are due to osteoporosis) will be excluded.

Heart valve replacements and mitral stenosis

Historically, AF patients have been classed as having valvular or non-valvular AF, but definitions of these have varied.[22] The licensing for DOACs in the UK states they should only be prescribed for patients with non-valvular AF, however, the randomised controlled trials for the DOACs all defined valvular AF differently and exclusion criteria varied.[23] Recently updated European Society of Cardiology guidelines suggest that VKAs should be the preferred option for patients with mechanical heart valves or moderate to severe mitral stenosis.[4] This co-variate has been included to investigate what proportion on DOAC and warfarin users have a history of mechanical heart valves or Read codes for moderate or severe mitral stenosis.

Number of GP visits

Number of GP visits in the year prior to study entry or the year preceding the OAC index date will be used as an indicator of healthcare utilisation.

Statistical analysis

Objective 1: To describe changes in the point prevalence and incidence of OAC prescribing by year prior to the introduction of DOACs (2003 – 2007), between the introduction of DOACs and the time they were recommended by NICE for stroke prevention in AF (2008 – 2012), and following NICE recommendation (2013 – 2017).

Point prevalence of OAC prescribing will be calculated at the midpoint of each year. Prescription mapping will be used to calculate the number of people with an OAC prescription that includes that time point as the numerator and the total number of people in the cohort at that time point as the denominator.

The incidence of OAC prescribing will be calculated overall and for each specific OAC. Everyone in the cohort will be considered 'at risk' until the time they receive their first OAC prescription. Incidence will be calculated using the number of incident OAC users as the numerator and person-time at risk for non-exposed persons as the denominator. For the person-time at risk calculation the denominator will be truncated at the date of their first OAC prescription. Incidence will be stratified by age (75-84 and 85+) and sex.

Objective 2: To describe switching between OACs during the study period

The number and percentage of patients switching from warfarin to DOAC, DOAC to DOAC and DOAC to warfarin will be calculated. The number of patients with multiple switches and details of the switches will be described. Average time to switch will be reported.

Objective 3: To compare the characteristics of patients with AF who were newly started on OACs during each period described in objective 1, to those who were not prescribed anticoagulation in the same period.

For peer review only - http://bmjopen.bmj.com/site/about/guidelines.xhtml

Demographics, characteristics, co-morbidities and concomitant medications will be reported for the three time periods described. Prevalent OAC users will be excluded from this part of the study as we want to compare those started on an OAC to those who are not during each time period.

Patient demographics (age, biological sex) and characteristics (body mass index (BMI), smoking status and alcohol consumption) will be reported as percentages in predefined categories. The proportion of the cohort with each of the co-morbidities and concomitant medications will be described. The mean CHA_2DS_2-VASc and HAS-BLED scores will be reported.

Objective 4: To describe persistence with DOACs compared with warfarin

Duration of prescribing of DOACs will be compared to warfarin using Cox-proportional hazards, stratified by DOAC and adjusted for age and other important covariates identified in objective 3. All incident OAC users will be included. Time on OAC will be calculated as the difference between the expected end date of the OAC and the start date, as defined above. Patients will be censored at the outcome (discontinuation of OAC therapy), on the date they stop contributing research standard data or the date of death.

All analyses will be conducted in Stata version 15.[24] All values will be reported with 95% confidence intervals.

Missing data

There is likely to be a substantial amount of missing date for BMI, smoking status and alcohol consumption as these variables may not be regularly updated by GP practices. Missing data will be assessed for randomness using a validated method and missing values imputed where appropriate to do so.

Patient and public involvement

Patients and the public were not involved in the production of this protocol or design of the study.

ETHICS AND DISSEMINATION

The protocol for this study was approved by the CPRD Independent Scientific Advisory Committee (ISAC; protocol 18_071) and is registered with the European Network of Centres for Pharmacoepidemiology and Pharmacovigilance (ENCePP; register number EUPAS29923). Results of the study will be disseminated in a peer-review journal with open access available within six months of publication. Results will also be communicated at national and international conferences as appropriate.

AUTHOR CONTRIBUTIONS

AM, TW MCW, JS and AMG all contributed to the design of the study. AM drafted the protocol which was then reviewed and revised by TW, JS and AMG.

FUNDING STATEMENT

This work was supported by The Dunhill Medical Trust [grant number RTF109/0117].

For peer review only - http://bmjopen.bmj.com/site/about/guidelines.xhtml

COMPETING INTERESTS

The authors declare no competing interests.

REFERENCES

1 Mant J, Hobbs FD, Fletcher K, et al. Warfarin versus aspirin for stroke prevention in an elderly community population with atrial fibrillation (the Birmingham Atrial Fibrillation Treatment of the Aged Study, BAFTA): a randomised controlled trial. *Lancet* 2007;370:493-503 doi:10.1016/s0140-6736(07)61233-1 [published Online First: 8 August 2007]

2 Pugh D, Pugh J, Mead GE. Attitudes of physicians regarding anticoagulation for atrial fibrillation: a systematic review. *Age Ageing* 2011;40:675-83 doi: 10.1093/ageing/afr097 [published Online First: 9 August 2009]

3 January CT, Wann LS, Calkins H, et al. 2019 AHA/ACC/HRS Focused Update of the 2014 AHA/ACC/HRS Guideline for the Management of Patients With Atrial Fibrillation: A Report of the American College of Cardiology/American Heart Association Task Force on Clinical Practice Guidelines and the Heart Rhythm Society. *J Am Coll Cardiol* 2019 Published Online First: 1 February 2019 doi: 10.1016/j.jacc.2019.01.011

4 Kirchhof P, Benussi S, Kotecha D, et al. 2016 ESC Guidelines for the management of atrial fibrillation developed in collaboration with EACTS. *Eur Heart J* 2016;37:2893-962 doi: 10.1093/eurheartj/ehw210 [published Online First: 27 August 2016]

5 National Institute for Health and Care Excellence (NICE). Atrial Fibrillation (CG180) 2014 [Available from: https://www.nice.org.uk/guidance/cg180 accessed 19th June 2019]

6 National Institute for Health and Care Excellence (NICE). TA249: Dabigatran etexilate for the prevention of stroke and systemic embolism in atrial fibrillation 2012 [Available from: https://www.nice.org.uk/guidance/ta249 accessed 19th June 2019]

7 National Institute for Health and Care Excellence (NICE). TA256: Rivaroxaban for the prevention of stroke and systemic embolism in people with atrial fibrillation 2012 [Available from: https://www.nice.org.uk/guidance/ta256 accessed 19th June 2019]

8 National Institute for Health and Care Excellence (NICE). TA275: Apixaban for preventing stroke and systemic embolism in people with nonvalvular atrial fibrillation 2013 [Available from: https://www.nice.org.uk/guidance/ta275 accessed 19th June 2019]

9 National Institute for Health and Care Excellence (NICE). TA355: Edoxaban for preventing stroke and systemic embolism in people with non-valvular atrial fibrillation 2015 [Available from: https://www.nice.org.uk/guidance/ta355 accessed 19th June 2019]

10 Connolly SJ, Ezekowitz MD, Yusuf S, et al. Dabigatran versus Warfarin in Patients with Atrial Fibrillation. *N Engl J Med* 2009;361:1139-51 doi:10.1056/NEJMoa0905561 [published Online First: 30 August 2009]

11 Patel MR, Mahaffey KW, Garg J, et al. Rivaroxaban versus Warfarin in Nonvalvular Atrial Fibrillation. *N Engl J Med* 2011;365:883-91 doi:10.1056/NEJMoa1009638 [published Online First: 10 August 2011]

12 Granger CB, Alexander JH, McMurray JJV, et al. Apixaban versus Warfarin in Patients with Atrial Fibrillation. *N Engl J Med* 2011;365:981-92 doi:10.1056/NEJMoa1107039 [published Online First: 28 August 2011]

For peer review only

For peer review only - http://bmjopen.bmj.com/site/about/guidelines.xhtml

13 Giugliano RP, Ruff CT, Braunwald E, et al. Edoxaban versus Warfarin in Patients with Atrial Fibrillation. *N Engl J Med* 2013;369:2093-104 doi:10.1056/NEJMoa1310907 [published Online First: 19 November 2013]

14 Gage BF, Birman-Deych E, Kerzner R, et al. Incidence of intracranial hemorrhage in patients with atrial fibrillation who are prone to fall. *Am J Med* 2005;118:612-7 doi:10.1016/j.amjmed.2005.02.022 [published Online First: 1 June 2005]

15 Loo SY, Dell'Aniello S, Huiart L, et al. Trends in the prescription of novel oral anticoagulants in UK primary care. *Br J Clin Pharmacol* 2017;83:2096-106 doi:10.1111/bcp.13299 [published Online First: 7 April 2017]

16 Herrett E, Gallagher AM, Bhaskaran K, et al. Data Resource Profile: Clinical Practice Research Datalink (CPRD). *Int J Epidemiol* 2015;44:827-36 doi:10.1093/ije/dyv098 [published Online First: 8 June 2015]

17 Lip GY, Nieuwlaat R, Pisters R, et al. Refining clinical risk stratification for predicting stroke and thromboembolism in atrial fibrillation using a novel risk factor-based approach: the euro heart survey on atrial fibrillation. *Chest* 2010;137:263-72 doi: 10.1378/chest.09-1584 [published Online First: 2009/09/19]

18 Pisters R, Lane DA, Nieuwlaat R, et al. A novel user-friendly score (HAS-BLED) to assess 1-year risk of major bleeding in patients with atrial fibrillation: the Euro Heart Survey. *Chest* 2010;138:1093-100. doi: 10.1378/chest.10-0134 [published Online First: 20 March 2010]

19 Schulman S, Kearon C. Definition of major bleeding in clinical investigations of antihemostatic medicinal products in non-surgical patients. *J Thromb Haemost* 2005;3:692-4 doi:10.1111/j.1538-7836.2005.01204.x [published Online First: 22 April 2005]

20 Kaatz S, Ahmad D, Spyropoulos AC, et al. Definition of clinically relevant non-major bleeding in studies of anticoagulants in atrial fibrillation and venous thromboembolic disease in non-surgical patients: communication from the SSC of the ISTH. *J Thromb Haemost* 2015;13:2119-26 doi:10.1111/jth.13140 [published Online First: 11 September 2015]

21 National Institute for Health and Care Excellence (NICE). CG146: Osteoporosis: assessing the risk of fragility fracture 2012 [Available from: https://www.nice.org.uk/guidance/cg146 accessed 18th April 2019].

22 Fauchier L, Philippart R, Clementy N, et al. How to define valvular atrial fibrillation? *Arch Cardiovasc Dis* 2015;108:530-9 doi:10.1016/j.acvd.2015.06.002 [published Online First: 18 July 2015]

23 Erwin JP, Iung B. Current recommendations for anticoagulant therapy in patients with valvular heart disease and atrial fibrillation: the ACC/AHA and ESC/EACTS Guidelines in Harmony…but not Lockstep! *Heart* 2018;104:968-70 doi: 10.1136/heartjnl-2017-312758 [published Online First: 11 January 2018]

24 Stata Statistical Software: Release 15 [program]. College Station, TX: StataCorp LP, 2017.

For peer review only

BMJ Open

Utilisation of oral anticoagulants in older people with atrial fibrillation in UK general practice: a protocol for a cohort study using the Clinical Practice Research Datalink (CPRD) database

Journal:	*BMJ Open*
Manuscript ID	bmjopen-2019-032646.R1
Article Type:	Protocol
Date Submitted by the Author:	22-Nov-2019

Complete List of Authors:	Mitchell, Anneka; University of Bath, Pharmacy and Pharmacology; University Hospital Southampton NHS Foundation Trust, Pharmacy Research Centre<br>Welsh, Tomas; Research Institute for the Care of Older People; University of Bristol, Institute of Clinical Neurosciences<br>Watson, Margaret; University of Strathclyde, Strathclyde Institute of Pharmacy and Biomedical Sciences<br>Snowball, Julia; University of Bath, Pharmacy and Pharmacology<br>McGrogan, Anita; University of Bath, Pharmacy and Pharmacology

**Primary Subject Heading**:	Geriatric medicine
Secondary Subject Heading:	Cardiovascular medicine, Epidemiology
Keywords:	Atrial fibrillation, Anticoagulants, Stroke < NEUROLOGY, Aged, Observational study

SCHOLARONE™
Manuscripts

UTILISATION OF ORAL ANTICOAGULANTS IN OLDER PEOPLE WITH ATRIAL FIBRILLATION IN UK GENERAL PRACTICE: A PROTOCOL FOR A COHORT STUDY USING THE CLINICAL PRACTICE RESEARCH DATALINK (CPRD) DATABASE

Anneka Mitchell[1, 2], Tomas Welsh[3, 4], Margaret C Watson[5], Julia Snowball[1] and Anita McGrogan[1]

1. Department of Pharmacy and Pharmacology, University of Bath, Bath, BA2 7AY, UK

2. Pharmacy Research Centre, University Hospital Southampton, Southampton, SO16 6YD, UK

3. Research Institute for the Care of Older People (RICE), Bath, BA1 3NG, UK

4. Institute of Clinical Neurosciences, University of Bristol, Bristol, BS8 1TH, UK

5. Strathclyde Institute of Pharmacy and Biomedical Sciences, University of Strathclyde, Glasgow, G4 0RE, UK

Corresponding author: Mrs Anneka Mitchell, Department of Pharmacy and Pharmacology, University of Bath, Bath, UK. BA2 7AY. Email: a.mitchell@bath.ac.uk Tel: 01225 384215

Word count: 5173

For peer review only

For peer review only - http://bmjopen.bmj.com/site/about/guidelines.xhtml

ABSTRACT

Introduction

Warfarin has frequently been under-used in older people for stroke prevention in atrial fibrillation (AF). Direct oral anticoagulants (DOACs) entered the UK market from 2008 and have been recommended as an alternative to warfarin. This study aims to describe any changes in the prescribing of OACs to people aged ≥ 75 years in UK general practice before and after the introduction of DOACs; examine differences in patient characteristics which may influence prescribers' decisions regarding anticoagulation; to evaluate the time people stay on OACs and switching between OACs.

Methods and analysis

A retrospective cohort study design will be used. Patients with a diagnosis of AF will be identified from the Clinical Practice Research Datalink (CPRD). The study period will run from 1st January 2003 to 27th December 2017. Patients enter the cohort at the latest date of: start of study period, first AF diagnosis, 75th birthday or a year from when they started to contribute research standard data. Follow-up continues until they leave the practice, death, the date the practice stops contributing research standard data or the end of the study period (27th December 2017). Exposure to OACs will be defined as ≥ 1 prescription issued for an OAC of interest during the study period. Patients entering the study on an OAC and those issued an OAC in the year preceding study entry will be defined as "prevalent users". Patients starting on an OAC during the study period will be defined as "incident users". Incidence and prevalence of OAC prescribing, patient demographics and characteristics will be described during three time periods: 2003-2007, 2008-2012, and 2013-2017. Persistence (defined as time from initiation to discontinuation of medication) with and switching between different OACs will be described.

Ethics and Dissemination

The protocol for this study was approved by the CPRD Independent Scientific Advisory Committee. The results will be disseminated in a peer-review journal and at conferences.

Trial registration number

EUPAS29923

Keywords

Atrial fibrillation, Anticoagulants, Stroke, Aged, Observational study

For peer review only - http://bmjopen.bmj.com/site/about/guidelines.xhtml

ARTICLE SUMMARY

Strengths and limitations of this study

- This will be a large cohort study using a validated data source which is representative of the UK population.
- This study will explore a number of aspects of OAC prescribing and examine changes over time to show any changes in OAC prescribing practices since the introduction of DOACs.
- Analysing differences in patient demographics, co-morbidities and co-prescribing between those prescribed DOACs and warfarin will provide important information for future studies aiming to compare differences in outcomes with the different OACs.
- This study relies on prescription data generated by general practices, therefore we will not know if the prescribed medication was ever dispensed or taken.
- Warfarin dosing schedules are not recorded in the CPRD so duration of warfarin prescriptions will be estimated.

INTRODUCTION

A large body of evidence has shown that vitamin K antagonists are safe and effective for stroke prevention in atrial fibrillation (AF), but they have frequently been under-used in older people.[1] Reasons cited by physicians as making them less likely to prescribe vitamin K antagonists for a person with AF include advancing age, risk of falls, co-morbidities and previous bleeding.[2]

The first direct oral anticoagulant (DOAC), dabigatran, was marketed throughout Europe and in the UK in 2008, but was not licensed for stroke prevention in AF until 2011. Since then, three additional DOACs have been licensed for prevention of stroke: rivaroxaban, apixaban and edoxaban, and these are recommended in national and international guidelines as an alternative to warfarin.[3-5] In the UK, the National Institute of Clinical Excellence (NICE) produces technology appraisals making recommendations for new medicines. The National Health Service (NHS) is legally obliged to fund those medicines recommended by NICE within 3 months of publication of the appraisal. NICE published favourable technology appraisals for dabigatran and rivaroxaban in 2012,[6 7] apixaban in 2013,[8] and edoxaban in 2015[9] meaning DOACs were available across the UK from mid-2012 onwards for stroke prevention in AF.

DOACs may allay some prescribers' concerns when deciding whether to anticoagulate their older patients: they have fixed dosing schedules and can be added to compliance aids. They have also been shown in clinical trials to have a significantly lower risk of intracranial haemorrhage than warfarin[10-13] (a prominent concern in patients with a history of falls).[14]

Since the introduction of DOACs, the overall rate of OAC initiation has increased by 58%.[15] However, it is not known whether the introduction of DOACs has changed the rates of OAC prescribing for older people (aged $\geq$ 75 years) or how patient demographics, co-

For peer review only

For peer review only - http://bmjopen.bmj.com/site/about/guidelines.xhtml

morbidities and concomitant prescribing affect the choice of anticoagulant prescribed in UK general practice.

This study aims to characterise prescribing of OACs to people aged ≥ 75 years in UK general practice, before and after the introduction of DOACs.

Objectives

1. To describe changes in the point prevalence and incidence of OAC prescribing by year prior to the introduction of DOACs (2003 – 2007), between the introduction of DOACs and the time they were recommended by NICE for stroke prevention in AF (2008 – 2012), and following NICE recommendation (2013 – 2017).

2. To describe switching between OACs during the study period.

3. To compare the characteristics of patients with AF who were newly started on OACs during each period described in objective 1, to those who were not prescribed anticoagulation in the same period.

4. To describe persistence with DOACs compared with warfarin.

METHODS AND ANALYSIS

This will be a retrospective cohort study using routinely collected healthcare data from the Clinical Practice Research Datalink (CPRD) database. The CPRD contains anonymised medical records and prescribing data from general practitioners in primary care. It contains data for around 7% of the UK population and is representative in age, sex and ethnicity.[16] Sociodemographic data, medical diagnoses, other clinical and test data are recorded using Read codes and these Read codes will be used to identify patients to be included in the study and also to identify covariates as described below.

Source population

The study period will start on the 1st January 2003 and end 27th December 2017. The source population will consist of all patients in CPRD who have ≥ 1 Read codes for AF and have contributed a minimum of 12 months' research standard data prior to entry in to the cohort. To be eligible to join the study, patients need to have the following: a Read code for AF recorded during or any time prior to the start of the study period; evidence to support the diagnosis of AF such as a second AF diagnosis at a later date, an echocardiogram or prescription for a rate limiting or antiarrhythmic medication in the 3 months before the AF diagnosis or any time after; and be aged ≥ 75 years at study entry.

Patients will enter the cohort at the latest of the following dates:

- Start of study period
- First AF diagnosis
- 75th birthday
- A year from the date they started to contribute research standard data

For peer review only - http://bmjopen.bmj.com/site/about/guidelines.xhtml

Patients will be censored if they leave the practice, the practice stops contributing data, at their date of death, or at the end of the study. For the incidence calculations and comparison of patient characteristics, patients will be censored when they are prescribed an OAC.

Time periods

The cohort will be analysed in three time periods:

- Period 1: prior to the introduction of DOACs (2003-2007)
- Period 2: during the period between the introduction of DOACs and the time they were recommended by NICE (2008 – 2012)
- Period 3: following the publication of the NICE technology appraisals recommending DOACs as an option for stroke prevention in AF (2013 – 2017).

For each time period, patients will contribute data to either the warfarin, DOAC or no OAC group. Patients in the cohort can contribute data to more than one time period and more than one group and may contribute differently for each objective. Patients may only contribute to the incident OAC group once with their index OAC. Any further prescriptions for different OACs would be considered to be prevalent.

Exposure

The OACs of interest in this study are warfarin, dabigatran, rivaroxaban, apixaban, and edoxaban. Warfarin is the only vitamin K antagonist (VKA) included as it is the most commonly used VKA in the UK. Dabigatran, rivaroxaban, apixaban and edoxaban are the only DOACs licensed for the prevention of stroke in AF available during the study period.

All prescriptions for the OACs of interest that are issued following an AF diagnosis, during the study period and in the year prior to entry in to the study cohort will be identified.

Incident and prevalent users

Exposure status will be defined at cohort entry as:

- "No OAC" where the patient had at least a year free of OAC prior to study entry and does not receive an OAC during the study period
- "Incident OAC users" where the patient had at least a year free of OAC prior to study entry then was prescribed an OAC during the study period
- "Prevalent OAC users" if the patient received an OAC in the year prior to study entry

Exposure status for incident OAC users will be stratified at the start of each study period to No OAC, warfarin or DOAC.

Indication for prescribing

OACs are prescribed for many different indications but only those prescribed for stroke prevention in AF will be included in this study. Patients will be excluded from the study if:

- They had a hip fracture or hip replacement in the 6 weeks prior to the OAC index date and only one prescription for an OAC
- They had a venous thromboembolism in the 6 months prior to the OAC index date

For peer review only - http://bmjopen.bmj.com/site/about/guidelines.xhtml

DOAC prescription duration

The index date will be defined as the date the first prescription was issued. The expected end date will be calculated by dividing the number of tablets prescribed by the licensed number of doses per day (i.e. 2 doses per day for dabigatran and apixaban and 1 dose per day for rivaroxaban and edoxaban). Where the quantity of tablets prescribed is not specified, the expected duration will be imputed from other prescriptions issued for the same product for that patient, or overall for that product if patient specific information is not available.

Patients will be classified as being continuously exposed until there is a gap of > 60 days between the expected end of one prescription and the start of the next. Where there is a gap of ≤ 60 days between the expected end of one prescription and the start of another for the same DOAC (regardless of whether the strength prescribed changes) the gap will be filled and defined as continuous exposure. Where a subsequent prescription for the same product is issued before the expected end of the previous prescription the prescriptions will be concatenated and the extra days added to the expected end date of the second prescription. A maximum of 60 additional days will allowed per patient.

A DOAC will be defined as discontinued 60 days from the expected end date of the last prescription issued for that product.

Warfarin prescription duration

Warfarin does not have a fixed dosing schedule; the dose is amended based on the results of a blood test, the international normalised ratio (INR). In the UK, patients are issued with different strength tablets (0.5mg, 1mg, 3mg and 5mg) and are advised, following each INR test, what dose they need to take and what strength of tablets to use. Dosing information for warfarin is not recorded in the CPRD, however, the strength and quantity of warfarin tablets issued by the general practice are recorded. Read codes for warfarin monitoring and results of INR tests are also recorded by some general practices but this is not recorded consistently.

The index date will be defined as either the date of the first prescription issued, or from the date of the first INR test result above 2 if this occurs before the first issue of a prescription. Patients may be exposed to warfarin before the first prescription issued by their general practitioner (GP) if treatment is started in hospital. CPRD data does not include hospital prescribing, hence INR test results ≥ 2 will be used as a surrogate marker of exposure.

The expected end date for warfarin prescriptions will be estimated using the median time between all previous prescriptions issued to the same patient. For example, if four prescriptions had been issued for 28 x 1mg tablets and the gaps between the prescription issue dates were 15, 35, and 42 days we would define the final prescription for 28 tablets as lasting 35 days so the expected end date of the last prescription would be 35 days from the issue date. Where multiple strengths are issued for a patient, the median will be calculated for each strength and the expected end date will be the latest of these dates.

If only one prescription has been issued the expected end date will be assumed to be equal to the median warfarin prescription gap for the cohort.

A patient will be assumed to have discontinued warfarin treatment if there is a period ≥ 2 times their median prescription gap. If an INR test result ≥ 2 is recorded in the discontinuation period (expected end date of last prescription to the date equal to twice their

For peer review only - http://bmjopen.bmj.com/site/about/guidelines.xhtml

median prescription gap), then INR tests will be used as a surrogate marker for warfarin prescribing and the end date will be defined as one week following the final INR test result ≥ 2.

The procedure for warfarin mapping is illustrated in Figure 1. This will be repeated for each strength and the patient classed as unexposed from the latest discontinuation date of all the strengths.

Restarting index OAC after discontinuation

There could be instances where OAC therapy is classed as discontinued when in fact the OAC has been continued but not issued by a GP if the patient is hospitalised. In instances where patients are classified as discontinuing OAC therapy but have then been "restarted" on their index OAC, they will be reviewed individually to ascertain whether the OAC therapy had truly been discontinued or whether their records indicate another reason for the gap in prescribing e.g. a record indicating an admission to hospital.

OAC Switching

The first OAC prescribed during the study (for incident users) or upon entry to the study (for prevalent users) will be defined as the index OAC. If ≥ 1 prescription for an alternative OAC is prescribed during the study period, the patient will be designated as having switched. Switches can be from:

- Warfarin to DOAC
- DOAC to warfarin
- DOAC to alternative DOAC

Patients will be assumed to have stopped the index OAC on the date the alternative OAC is issued if this is prior to the expected end date of the index OAC. Where the alternative OAC is prescribed after the expected end date of the index OAC, the time to switch will be described.

Covariate classification

It is recognised that some patients may remain in the cohort for a long time and their covariates may change. Therefore covariates will be identified separately for each of the time periods the patient contributes data and be grouped by DOAC, warfarin or no OAC depending on exposure for that period.

We want to investigate whether certain comorbidities influence the prescribing of OACs. We will therefore look for comorbidities accumulated up to the end of the time period for patients who do not receive an OAC or the OAC index date where one is prescribed. Patients will be classified as having a co-morbidity if the patient record contains ≥ 1 diagnostic Read code for a disease or ≥ 3 Read codes supporting the presence of the disease at any time prior to the relevant date for that period. Read codes used to support the presence of disease include attendance at specialist clinics (e.g. diabetes clinics) or codes related to follow up such as hypertension monitoring.

For peer review only - http://bmjopen.bmj.com/site/about/guidelines.xhtml

Concomitant medication will be classified as ≥ 1 prescription issues for a medication of interest in the 3 months prior to study entry, commencing an OAC, or the end of the time period. We will describe medications at study entry as baseline and then in each time period for the no OAC and incident OAC patients as described for the comorbidities above. The medications of interest either affect stroke or bleeding risk or commonly interact with OACs (antihypertensives, antiplatelets, statins, diabetic medications, antiarrythmics, NSAIDs, corticosteroids, anticonvulsants, systemic azoles, HIV protease inhibitors, and ciclosporin).

Definitions of covariates

Differences in patient demographics (age, biological sex) and characteristics (body mass index (BMI), smoking status and alcohol consumption) between those anticoagulated for AF and those not anticoagulated will be described for each period. Differences in the stroke risk score (CHA_2DS_2-VASc)[17] and the bleeding risk score (HAS-BLED),[18] co-morbidities and concomitant medication will also be described. These covariates are defined in the following subsections.

CHA_2DS_2-VASc score

International guidelines recommend using this score to calculate stroke risk in patients with AF.[3-5] Higher scores indicate an increasing risk of stroke and the maximum score is 9. Definitions for each element were based on those described by Lip and colleagues[17] but have been modified for clarity:

- *Congestive heart failure (1 point)*
  Right or left ventricular failure or heart failure. Supportive information includes Read codes for heart failure clinic attendance or referral, heart failure monitoring or reference to heart failure therapy/education.
- *Hypertension (1 point)*
  Primary or secondary hypertension or hypertensive disease states e.g. hypertensive heart disease. Supportive information includes codes for monitoring, referral to specialist clinics or reference to therapy for hypertension.
- *Age ≥ 75 (2 points)*
- *Diabetes mellitus (1 point)*
  Type 1 or Type 2 diabetes mellitus diagnoses or the consequences of diabetes mellitus e.g. diabetic retinopathy. This also includes anyone prescribed oral hypoglycaemic agents and/or insulin. Excludes gestational diabetes and steroid induced diabetes as these are often reversible. Supportive information includes dietary advice, reference to diabetes therapies, referral to specialist clinics or educational programs.
- *Stroke/transient ischaemic attack or thromboembolism (2 points)*
  Stroke: Includes ischaemic stroke transient ischaemic attacks. Excludes haemorrhagic stroke, and venous infarctions.
  Thromboembolism: Includes arterial embolic disease and surgeries to treat arterial emboli, excludes thrombosis and venous embolic disease which are included in vascular disease.
- *Vascular disease (1 point)*
  Includes: Coronary artery disease (prior myocardial infarction, angina pectoris, percutaneous coronary intervention or coronary bypass surgery and aortic plaque);

For peer review only - http://bmjopen.bmj.com/site/about/guidelines.xhtml

Peripheral vascular disease (intermittent claudication, previous surgery or percutaneous intervention on the abdominal aorta, bifurcation or the lower extremity vessels (limited to surgery on the femoral, popliteal, peroneal and iliac arteries)); arterial and venous thrombosis. Excludes thromboses related to pregnancy.

- *Age 64-74 (1 point)*
- *Female sex (score 1)*

HAS-BLED [18]

International guidelines recommend using this score to assess the risk of bleeding with anticoagulants.[3-5] Higher scores indicate an increased risk of bleeding with anticoagulants. The presence of each disease scores 1 point and the maximum score is 9. For the purpose of this study, the maximum score for the HAS-BLED will be 8. The L in HAS-BLED corresponds to labile INR defined as a time in therapeutic range with warfarin of less than 60%. As INRs are not recorded consistently in the CPRD and because people prescribed DOACs would not have INR tests at all, this element of the score will not be included. Definitions for each element were based on those described by Pisters and colleagues,[18] but have been adapted to suit the information recorded in the CPRD.

- *Hypertension (1 point)*
  As described for CHA_2DS_2-VASc score above
- *Abnormal renal or hepatic function (1 point each, max 2 points)*
  Renal and hepatic function will be determined using a combination of clinical Read codes and test results.
  - Renal impairment:
    Includes chronic dialysis, transplant, chronic renal impairment (where defined as stage 3 or above, moderate, severe or end-stage). If renal impairment is recorded with no severity, but there is a test result for creatinine $\geq$ 200µmol/L the patient will be allocated a point. If there are no test results recorded the patient will score zero. Codes for renal disease where it does not state that renal function is impaired will be excluded.
  - Hepatic disease:
    Includes chronic hepatic disease (cirrhosis, hepatitis, fatty liver, and fibrosis), liver transplantation, or biochemical evidence of significant hepatic derangement (bilirubin > 2 x upper limit of normal in association with aspartate aminotransferase/alanine aminotransferase/alkaline phosphatase) where these blood test results are recorded in CPRD.
- *Stroke (1 point)*
  As described for CHA_2DS_2-VASc score above
- *Bleeding history or predisposition (1 point)*
  Includes any previous bleed. Categorised based on the International Society of Thrombosis and Haemostasis guidance as major bleeding where the bleed occurs in a critical area or organ or requires a blood transfusion (CPRD does not state the number of units infused so any requirement for a blood transfusion in conjunction with a Read code for bleeding will be classified as a major bleed);[19] or clinically relevant non major bleeding (defined as a bleed not fitting the major bleed criteria but requiring review by a healthcare professional, as the codes are recorded by a GP we will assume

For peer review only - http://bmjopen.bmj.com/site/about/guidelines.xhtml

that these bleeds required review).[20] Excludes codes for traumatic bleeding, bleeding related to pregnancy and post-operative bleeding. Predisposition to bleeding includes anaemia and gastric, duodenal and peptic ulcers.

- *Elderly (1 point)*
  Defined as being aged > 65 years old
- *Drugs or Alcohol (1 point each, max 2 points)*
  A point will be allocated where a patient receives a prescription for an antiplatelet or non-steroidal anti-inflammatory (NSAID) medication within the time period. Exact alcohol consumption is not well documented in the CPRD but patients will score a point for this element if they have any record stating the number of units consumed per week as > 8 units or if they have any codes for heavy/problem drinking.

Renal Impairment

DOACs are predominantly renally cleared and so require dose adjustment and careful monitoring in renal impairment. The elimination of warfarin is mostly metabolic, being metabolised by hepatic enzymes so is not affected by changes in renal function. Renal impairment may therefore affect the decision of which anticoagulant (if any) to use so has been included as a covariate alone, in addition to its inclusion in the HAS-BLED score. The Read code or test result dated closest to the relevant date for the period will be used to categorise the presence and level of renal impairment.

- *Chronic renal impairment*
  Includes chronic dialysis, renal impairment, and chronic kidney disease regardless of whether severity or stage is reported. Test results for creatinine and estimated glomerular filtration rate (eGFR) will also be obtained where available. If test results are available, they will be used to classify whether a patient has renal impairment and the stage of impairment (if eGFR is documented). Codes for a renal disease that may reduce kidney function but with no test or diagnostic code to support the presence of renal impairment will be excluded.
- *Acute kidney injury*
  Previous episodes of acute kidney injury may influence the decision to prescribe an anticoagulant, and which anticoagulant to prescribe. Read codes for acute kidney injury will be counted as discrete events where they occur $\geq$ 1 week apart.

Hepatic disease

Hepatic disease is an independent risk factor for bleeding and also affects the metabolism of warfarin so may affect the decision to prescribe anticoagulation. Hepatic disease will be defined as specified under HAS-BLED above.

Previous bleed

Includes any non-traumatic bleeding or haemorrhage. Bleeds will be divided in to subsections of major bleeding and clinically relevant non major bleeding (as described in HAS-BLED section), and also by site of bleed (intracranial bleeds, gastrointestinal bleeds, other).

For peer review only - http://bmjopen.bmj.com/site/about/guidelines.xhtml

Dementia

Include dementia (any type), Alzheimer's disease, and anyone prescribed an acetylcholinesterase inhibitor or memantine. Excludes cognitive impairment where there is no diagnosis of dementia.

History of or at risk of falls

Includes any type of fall and any Read code which specifically indicates the practitioner is concerned that the patient is at risk of falling. Excludes gait disturbances and other factors which may increase risk of falling but have not been defined by the GP as causing concern regarding falls risk.

Fragility fractures

Fragility fractures are defined by the National Institute of Clinical Excellence (NICE) as "…most commonly in the spine (vertebrae), hip (proximal femur) and wrist (distal radius). They may also occur in the arm (humerus), pelvis, ribs and other bones".[21] It is often not recorded in CPRD whether a fracture is thought to be a fragility fracture or not. Read codes for a diagnosis of fracture or operation to repair a fracture occurring in the spine, proximal femur, long bones, distal radius, humerus, pelvis or ribs will be included. Non-specific codes for osteoporotic and fragility fractures will also be included. Pathological fractures (unless specified that they are due to osteoporosis) will be excluded.

Heart valve replacements and mitral stenosis

Historically, AF patients have been classed as having valvular or non-valvular AF, but definitions of these have varied.[22] The licensing for DOACs in the UK states they should only be prescribed for patients with non-valvular AF, however, the randomised controlled trials for the DOACs all defined valvular AF differently and exclusion criteria varied.[23] Recently updated European Society of Cardiology guidelines suggest that VKAs should be the preferred option for patients with mechanical heart valves or moderate to severe mitral stenosis.[4] This co-variate has been included to investigate what proportion on DOAC and warfarin users have a history of mechanical heart valves or Read codes for moderate or severe mitral stenosis.

Number of general practice (GP) encounters

The number of encounters with healthcare professionals from general practice in the year prior to study entry, the year preceding the OAC index date or preceding the end of the time period will be used as an indicator of healthcare utilisation.

Statistical analysis

Objective 1: To describe changes in the point prevalence and incidence of OAC prescribing by year prior to the introduction of DOACs (2003 – 2007), between the introduction of DOACs and the time they were recommended by NICE for stroke prevention in AF (2008 – 2012), and following NICE recommendation (2013 – 2017).

Only patients prescribed an OAC will contribute data to the incidence and prevalence calculations. For incidence, patients may only contribute to either the warfarin or DOAC group once as they will be censored at the time they are prescribed an OAC. The point

For peer review only - http://bmjopen.bmj.com/site/about/guidelines.xhtml

prevalence will be calculated at the mid-point of each year so will take account of switching. For prevalence, a patient could contribute to the warfarin group for some years, and the DOAC group for other years. Prescription mapping will be used to calculate the number of people with an OAC prescription that includes that time point as the numerator and the total number of people in the cohort at that time point as the denominator.

The incidence of OAC prescribing will be calculated overall and for each specific OAC. Everyone in the cohort will be considered 'at risk' until the time they receive their first OAC prescription. Incidence will be calculated using the number of incident OAC users as the numerator and person-time at risk for non-exposed persons as the denominator. For the person-time at risk calculation the denominator will be truncated at the date of their first OAC prescription. Incidence will be stratified by age (75-84 and 85+) and sex.

Figure 2 illustrates how patients may contribute to the incidence and prevalence calculations throughout the study.

Objective 2: To describe switching between OACs during the study period

The number and percentage of patients switching from warfarin to DOAC, DOAC to DOAC and DOAC to warfarin will be calculated. The number of patients with multiple switches and details of the switches will be described. Average time to switch will be reported.

Objective 3: To compare the characteristics of patients with AF who were newly started on OACs during each period described in objective 1, to those who were not prescribed anticoagulation in the same period.

Demographics, characteristics, co-morbidities and concomitant medications will be reported for the three time periods described. Prevalent OAC users will be excluded from this part of the study as we want to compare those started on an OAC to those who are not during each time period.

Patients may contribute data to more than one group but they will be censored at the time they are prescribed their index OAC. For example they may enter the study during the first time period but not be prescribed an OAC until period 3. In this case they would contribute data to the no-OAC group in time period 1 and 2 and then one of the OAC groups in period 3. If they entered in period 1 and started on an OAC in period 2, they would contribute data to the no OAC group in period 1 and the OAC group in period 2, they would not contribute any data to period 3 as they would be censored. Figure 3 illustrates how patients contribute to different groups during their time in the study. Patient demographics (age, biological sex) and characteristics (body mass index (BMI), smoking status and alcohol consumption) will be reported as percentages in predefined categories. The proportion of the cohort with each of the co-morbidities and concomitant medications will be described. The mean CHA_2DS_2-VASc and HAS-BLED scores will be reported.

Objective 4: To describe persistence with DOACs compared with warfarin

Duration of prescribing of DOACs will be compared to warfarin using Cox-proportional hazards, stratified by DOAC and adjusted for age and other important covariates identified in objective 3. All incident OAC users will be included. Time on OAC will be calculated as the difference between the discontinuation date of the OAC and the index date, as defined above.

For peer review only - http://bmjopen.bmj.com/site/about/guidelines.xhtml

Patients will be censored at the outcome (discontinuation of OAC therapy), on the date they stop contributing research standard data or the date of death.

All analyses will be conducted in Stata version 15.[24] All values will be reported with 95% confidence intervals.

Missing data

There is likely to be a substantial amount of missing data for weight, BMI, smoking status and alcohol consumption as these variables may not be regularly updated by GP practices. We will use logistic regression to ascertain whether other variables (age, sex, or comorbidities) predict whether these data are missing. If these variables do not predict the missing data then we will assume that the data is missing at random. The proportion of missing data for these variables will be presented. If the data is missing at random we will impute the missing values using multiple imputation and conduct a sensitivity analysis to investigate the effect of the missing data on the results of the analyses. However, previous studies suggest it may only be appropriate to impute weight and BMI. [25,26]

Patient and public involvement

Patients and the public were not involved in the production of this protocol or design of the study.

DISCUSSION

A number of studies have described changes in anticoagulation prescribing in the time since DOACs were introduced in the UK.[15,27,28] However, none have focussed specifically on their use in older people aged ≥ 75 years, despite older people being the largest users of anticoagulants for AF. The main strengths of this study are that it will investigate prescribing in a large cohort of older patients using the CPRD which is generally representative of the UK population. This study will look at a number of different factors that may influence the prescribing of anticoagulants and how these have changed over time. The results of this study will inform future comparative studies between OACs as it will provide an insight to potential confounders which may need to be controlled for. A limitation of this study is defining accurately when patients are exposed to an anticoagulant and when they are not. This is particularly difficult for warfarin as dosing information is not included in the CPRD. A number of studies have compared DOACs and warfarin using routinely collected healthcare data but they do not describe how they mapped warfarin exposure. [29,30] This protocol is transparent in describing our planned method for warfarin mapping and uses both prescription and test result data. We will also investigate gaps in prescribing to establish whether patients may have been in hospital or having their OAC prescribed elsewhere, this is likely to be more common in this age group than others and has been noted as a limitation in other studies.[31] A second limitation is that comorbidities may not always be recorded in the CPRD, particularly where they are diagnosed and treated in secondary care. However, as the comorbidities we have chosen are chronic and likely to have a significant impact on patients' health, it is likely that they would be recorded accurately. Finally, as it is difficult to predict whether prescriptions for concomitant medicines are still continuing at any given time, we have decided to assume that if a prescription was issued in the three months prior to an OAC being started that the concomitant medicine is still continuing at that time. This is likely to be

For peer review only - http://bmjopen.bmj.com/site/about/guidelines.xhtml

the case as most medications of interest are for chronic conditions. However, we acknowledge that it may overestimate exposure to these concomitant medications.

ETHICS AND DISSEMINATION

The protocol for this study was approved by the CPRD Independent Scientific Advisory Committee (ISAC; protocol 18_071) and is registered with the European Network of Centres for Pharmacoepidemiology and Pharmacovigilance (ENCePP; register number EUPAS29923). Results of the study will be disseminated in a peer-review journal with open access available within six months of publication. Results will also be communicated at national and international conferences as appropriate.

AUTHOR CONTRIBUTIONS

AM, TW, MCW, JS and AMG all contributed to the design of the study. AM drafted the protocol which was then reviewed and revised by TW, JS, MCW and AMG.

FUNDING STATEMENT

This work was supported by The Dunhill Medical Trust [grant number RTF109/0117].

COMPETING INTERESTS

The authors declare no competing interests.

REFERENCES

1 Mant J, Hobbs FD, Fletcher K, et al. Warfarin versus aspirin for stroke prevention in an elderly community population with atrial fibrillation (the Birmingham Atrial Fibrillation Treatment of the Aged Study, BAFTA): a randomised controlled trial. *Lancet* 2007;370:493-503 doi:10.1016/s0140-6736(07)61233-1 [published Online First: 8 August 2007]

2 Pugh D, Pugh J, Mead GE. Attitudes of physicians regarding anticoagulation for atrial fibrillation: a systematic review. *Age Ageing* 2011;40:675-83 doi: 10.1093/ageing/afr097 [published Online First: 9 August 2009]

3 January CT, Wann LS, Calkins H, et al. 2019 AHA/ACC/HRS Focused Update of the 2014 AHA/ACC/HRS Guideline for the Management of Patients With Atrial Fibrillation: A Report of the American College of Cardiology/American Heart Association Task Force on Clinical Practice Guidelines and the Heart Rhythm Society. *J Am Coll Cardiol* 2019 Published Online First: 1 February 2019 doi: 10.1016/j.jacc.2019.01.011

4 Kirchhof P, Benussi S, Kotecha D, et al. 2016 ESC Guidelines for the management of atrial fibrillation developed in collaboration with EACTS. *Eur Heart J* 2016;37:2893-962 doi: 10.1093/eurheartj/ehw210 [published Online First: 27 August 2016]

5 National Institute for Health and Care Excellence (NICE). Atrial Fibrillation (CG180) 2014 [Available from: https://www.nice.org.uk/guidance/cg180 accessed 19th June 2019]

6 National Institute for Health and Care Excellence (NICE). TA249: Dabigatran etexilate for the prevention of stroke and systemic embolism in atrial fibrillation 2012 [Available from: https://www.nice.org.uk/guidance/ta249 accessed 19th June 2019]

For peer review only - http://bmjopen.bmj.com/site/about/guidelines.xhtml

7 National Institute for Health and Care Excellence (NICE). TA256: Rivaroxaban for the prevention of stroke and systemic embolism in people with atrial fibrillation 2012 [Available from: https://www.nice.org.uk/guidance/ta256 accessed 19th June 2019]

8 National Institute for Health and Care Excellence (NICE). TA275: Apixaban for preventing stroke and systemic embolism in people with nonvalvular atrial fibrillation 2013 [Available from: https://www.nice.org.uk/guidance/ta275 accessed 19th June 2019]

9 National Institute for Health and Care Excellence (NICE). TA355: Edoxaban for preventing stroke and systemic embolism in people with non-valvular atrial fibrillation 2015 [Available from: https://www.nice.org.uk/guidance/ta355 accessed 19th June 2019]

10 Connolly SJ, Ezekowitz MD, Yusuf S, et al. Dabigatran versus Warfarin in Patients with Atrial Fibrillation. *N Engl J Med* 2009;361:1139-51 doi:10.1056/NEJMoa0905561 [published Online First: 30 August 2009]

11 Patel MR, Mahaffey KW, Garg J, et al. Rivaroxaban versus Warfarin in Nonvalvular Atrial Fibrillation. *N Engl J Med* 2011;365:883-91 doi:10.1056/NEJMoa1009638 [published Online First: 10 August 2011]

12 Granger CB, Alexander JH, McMurray JJV, et al. Apixaban versus Warfarin in Patients with Atrial Fibrillation. *N Engl J Med* 2011;365:981-92 doi:10.1056/NEJMoa1107039 [published Online First: 28 August 2011]

13 Giugliano RP, Ruff CT, Braunwald E, et al. Edoxaban versus Warfarin in Patients with Atrial Fibrillation. *N Engl J Med* 2013;369:2093-104 doi:10.1056/NEJMoa1310907 [published Online First: 19 November 2013]

14 Gage BF, Birman-Deych E, Kerzner R, et al. Incidence of intracranial hemorrhage in patients with atrial fibrillation who are prone to fall. *Am J Med* 2005;118:612-7 doi:10.1016/j.amjmed.2005.02.022 [published Online First: 1 June 2005]

15 Loo SY, Dell'Aniello S, Huiart L, et al. Trends in the prescription of novel oral anticoagulants in UK primary care. *Br J Clin Pharmacol* 2017;83:2096-106 doi:10.1111/bcp.13299 [published Online First: 7 April 2017]

16 Herrett E, Gallagher AM, Bhaskaran K, et al. Data Resource Profile: Clinical Practice Research Datalink (CPRD). *Int J Epidemiol* 2015;44:827-36 doi:10.1093/ije/dyv098 [published Online First: 8 June 2015]

17 Lip GY, Nieuwlaat R, Pisters R, et al. Refining clinical risk stratification for predicting stroke and thromboembolism in atrial fibrillation using a novel risk factor-based approach: the euro heart survey on atrial fibrillation. *Chest* 2010;137:263-72 doi: 10.1378/chest.09-1584 [published Online First: 2009/09/19]

18 Pisters R, Lane DA, Nieuwlaat R, et al. A novel user-friendly score (HAS-BLED) to assess 1-year risk of major bleeding in patients with atrial fibrillation: the Euro Heart Survey. *Chest* 2010;138:1093-100. doi: 10.1378/chest.10-0134 [published Online First: 20 March 2010]

19 Schulman S, Kearon C. Definition of major bleeding in clinical investigations of antihemostatic medicinal products in non-surgical patients. *J Thromb Haemost* 2005;3:692-4 doi:10.1111/j.1538-7836.2005.01204.x [published Online First: 22 April 2005]

20 Kaatz S, Ahmad D, Spyropoulos AC, et al. Definition of clinically relevant non-major bleeding in studies of anticoagulants in atrial fibrillation and venous thromboembolic disease

For peer review only - http://bmjopen.bmj.com/site/about/guidelines.xhtml

in non-surgical patients: communication from the SSC of the ISTH. *J Thromb Haemost* 2015;13:2119-26 doi:10.1111/jth.13140 [published Online First: 11 September 2015]

21 National Institute for Health and Care Excellence (NICE). CG146: Osteoporosis: assessing the risk of fragility fracture 2012 [Available from: https://www.nice.org.uk/guidance/cg146 accessed 18th April 2019].

22 Fauchier L, Philippart R, Clementy N, et al. How to define valvular atrial fibrillation? *Arch Cardiovasc Dis* 2015;108:530-9 doi:10.1016/j.acvd.2015.06.002 [published Online First: 18 July 2015]

23 Erwin JP, Iung B. Current recommendations for anticoagulant therapy in patients with valvular heart disease and atrial fibrillation: the ACC/AHA and ESC/EACTS Guidelines in Harmony…but not Lockstep! *Heart* 2018;104:968-70 doi: 10.1136/heartjnl-2017-312758 [published Online First: 11 January 2018]

24 Stata Statistical Software: Release 15 [program]. College Station, TX: StataCorp LP, 2017.

25 Bhaskaran K, Forbes HJ, Douglas I, et al. Representativeness and optimal use of body mass index (BMI) in the UK Clinical Practice Research Datalink (CPRD). *BMJ Open* 2013;3: p. e003389 doi: 10.1136/bmjopen-2013-003389

26 Marston L, Carpenter JR, Walters KR, et al. Issues in multiple imputation of missing data for large general practice clinical databases. *Pharmacoepidemiol Drug Saf* 2010;19: 618-26 doi: 10.1002/pds.1934

27 Adderley NJ, Ryan R, Nirantharakumar K, et al. Prevalence and treatment of atrial fibrillation in UK general practice from 2000 to 2016. *Heart* 2019;105:27-33 doi: 10.1136/heartjnl-2018-312977

28 Lacoin L, Lumley M, Ridha E, et al. Evolving landscape of stroke prevention in atrial fibrillation within the UK between 2012 and 2016: a cross-sectional analysis study using CPRD. *BMJ Open* 2017;7:e015363 doi: 10.1136/bmjopen-2016-015363

29 Vinogradova Y, Coupland C, Hill T, et al. Risks and benefits of direct oral anticoagulants versus warfarin in a real world setting: cohort study in primary care. *BMJ* 2018;362:k2505 doi: 10.1136/bmj.k2505

30 Loo SY, Coulombe J, Dell'Aniello S. Comparative effectiveness of novel oral anticoagulants in UK patients with non-valvular atrial fibrillation and chronic kidney disease: a matched cohort study. *BMJ Open* 2018;8: e019638 doi: 10.1136/bmjopen-2017-019638

31 Mueller T, Alvarez-Madrazo S, Robertson C. Use of direct oral anticoagulants in patients with atrial fibrillation in Scotland: Applying a coherent framework to drug utilisation studies. *Pharmacoepidemiol Drug Saf* 2017;26:1378-1386 doi: 10.1002/pds.4272

FIGURE LEGENDS

Figure 1. Illustration on warfarin mapping for a single patient and one strength of warfarin tablet. Rx = prescription

Figure 2. Illustration showing how patients may contribute to the different groups for incidence and prevalence. The blue arrows show total unexposed time in study, X = left the study, red arrow = exposed to warfarin, purple arrow = exposed to DOAC. The vertical arrows show the yearly incidence and prevalence calculations and the letters in boxes

For peer review only - http://bmjopen.bmj.com/site/about/guidelines.xhtml

correspond to the group the patient would contribute to for that year. D = denominator (incidence and prevalence for first D then prevalence only for all subsequent D), I = incidence numerator, P = prevalence numerator.

Figure 3. Illustration showing how patients contribute to different groups for the characteristic comparisons during their time in the study. Blue arrows represent individual patients, the stars represent the date the index OAC was commenced, X represents the patient leaving the study. The boxes describe which group the patient will contribute comorbidity data to for that period (OAC will be subdivided to warfarin or DOAC depending which was prescribed).

For peer review only

For peer review only - http://bmjopen.bmj.com/site/about/guidelines.xhtml

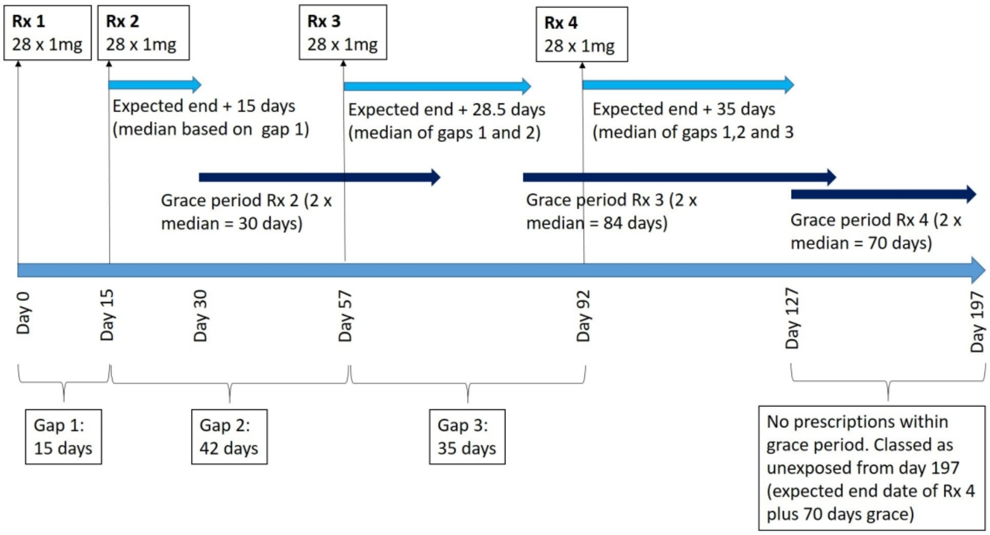

Figure 1. Illustration on warfarin mapping for a single patient and one strength of warfarin tablet. Rx = prescription

165x88mm (300 x 300 DPI)

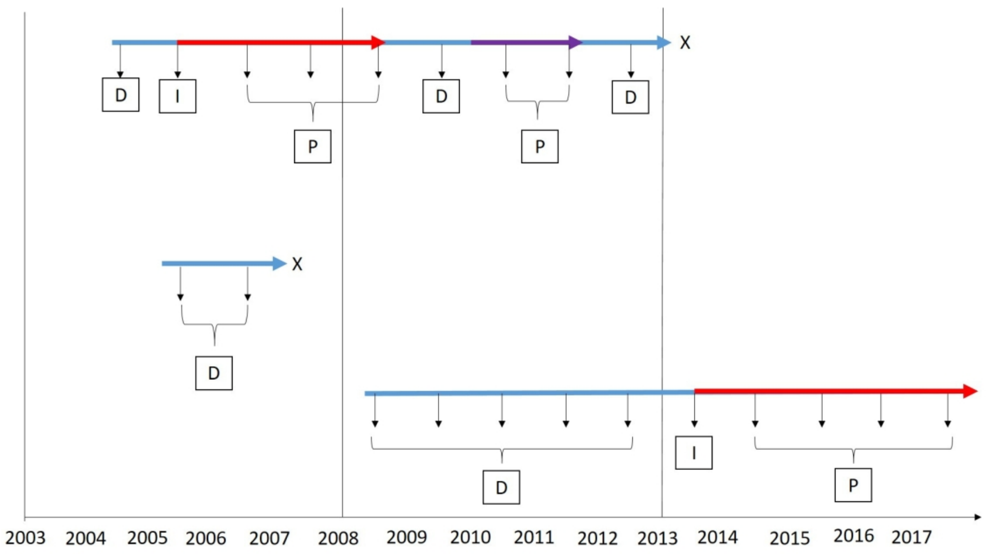

Figure 2. Illustration showing how patients may contribute to the different groups for incidence and prevalence. The blue arrows show total unexposed time in study, X = left the study, red arrow = exposed to warfarin, purple arrow = exposed to DOAC. The vertical arrows show the yearly incidence and prevalence calculations and the letters in boxes correspond to the group the patient would contribute to for that year. D = denominator (incidence and prevalence for first D then prevalence only for all subsequent D), I = incidence numerator, P = prevalence numerator.

156x86mm (300 x 300 DPI)

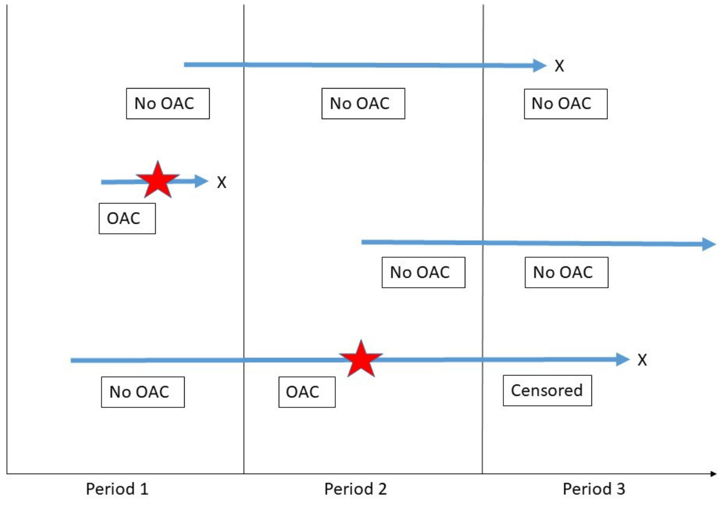

Figure 3. Illustration showing how patients contribute to different groups for the characteristic comparisons during their time in the study. Blue arrows represent individual patients, the stars represent the date the index OAC was commenced, X represents the patient leaving the study. The boxes describe which group the patient will contribute comorbidity data to for that period (OAC will be subdivided to warfarin or DOAC depending which was prescribed).

108x60mm (300 x 300 DPI)
